# Antitumor Activity of Monoterpenes Found in Essential Oils

**DOI:** 10.1155/2014/953451

**Published:** 2014-10-14

**Authors:** Marianna Vieira Sobral, Aline Lira Xavier, Tamires Cardoso Lima, Damião Pergentino de Sousa

**Affiliations:** ^1^Departamento de Ciências Farmacêuticas, Universidade Federal da Paraíba, 58051-970 João Pessoa, PB, Brazil; ^2^Programa de Pós-Graduação em Produtos Naturais e Sintéticos Bioativos, Universidade Federal da Paraíba, Caixa Postal 5009, 58015-970 João Pessoa, PB, Brazil; ^3^Departamento de Farmácia, Universidade Federal de Sergipe, 49100-000 São Cristóvão, SE, Brazil

## Abstract

Cancer is a complex genetic disease that is a major public health problem worldwide, accounting for about 7 million deaths each year. Many anticancer drugs currently used clinically have been isolated from plant species or are based on such substances. Accumulating data has revealed anticancer activity in plant-derived monoterpenes. In this review the antitumor activity of 37 monoterpenes found in essential oils is discussed. Chemical structures, experimental models, and mechanisms of action for bioactive substances are presented.

## 1. Introduction

Cancer is a complex genetic disease that comprises specific hallmarks. They include sustaining proliferative signaling, evading growth suppressors, resisting cell death, enabling replicative immortality, inducing angiogenesis, and activating invasion and metastasis, apart from reprogramming of energy metabolism and evading immune destruction [1]. According to the World Health Organization (WHO), the overall impact of cancer has increased by more than the double in the last 30 years. It is estimated that in 2008 there were roughly 12 million new cancer cases and seven million deaths worldwide. Future projections indicate that cancer mortality will continue to rise, reaching 11.4 million in 2030 [2]. The study of natural products has been the single most successful strategy for the discovery of new medicines used in anticancer therapy, and more than two thirds of the drugs used in cancer treatment come directly from natural products or are developed using knowledge gained from the activity of their ingredients [3, 4].

In these recent years, a large number of studies have documented the efficacy of essential oils and their chemical constituents as source of new bioactive natural products [5], including against cancer [6, 7]. For example, Piaru and collaborators [8] investigated the cytotoxicity of the essential oils from* Myristica fragrans* and* Morinda citrifolia.* The results showed that the* M. fragrans* essential oil exhibited greater cytotoxic activity than the* M. citrifolia* oil, possibly due to the presence of some potential anticancer substances such as limonene, terpinen-4-ol, eugenol, and myristicin. In another study, Ferraz and collaborators [9] revealed the cytotoxic effect of leaf essential oil of* Lippia gracilis* Schauer and its constituents (thymol,* p*-cymene, *γ*-terpinene, and myrcene). Interestingly, Maggi and collaborators [10] investigated the antiproliferative activity of essential oil from* Vepris macrophylla.* This oil demonstrated a strong cytotoxic effect, which may be attributed by the presence of specific components such as the monoterpenes citral, citronellol, and myrcene. Furthermore, Nikolić and collaborators [11] investigated the antitumor activity of* Thymus serpyllum, T. algeriensis,* and* T. vulgaris* essential oils on growth of four human tumor cells. The specie* T. serpyllum* was the most potent in all tested cell lines and contains thymol as its major constituent, a phenolic compound known in the literature for its antiproliterative activity [12]. Therefore, the essential oils and chemical constituents are natural products with high pharmacological potential against various types of tumors.

Cancer is a major cause of death worldwide, ranked behind only cardiovascular disease. Considering that monoterpenes are common in many plant species and are used in cosmetic and pharmaceutical preparations, as well as the food industry, it is important to review the pharmacological potential of monoterpenes with anticancer activity.

The present study was carried out based on the literature review of monoterpenes from essential oils with antitumor activity. Chemical structures and names of bioactive compounds are provided. The monoterpenes presented in this review were selected with reference to effects shown in specific experimental models for evaluation of antitumor activity and/or by complementary studies aimed to elucidate mechanisms of action Table 1. The selection of essential oils constituents in the database was related to various terms, including essential oils and monoterpenes as well as names of representative compounds of these chemical groups refining with antitumor activity, cytotoxic activity, and cytotoxicity. The search was performed in the scientific literature databases and Chemical Abstracts in September 2013.

### 1.1. Linalyl Acetate, Alpha-Terpineol, and Camphor

Linalyl acetate, alpha-terpineol, and camphor in association linalyl acetate, alpha-terpineol, and camphor caused inhibition of the growth of the human colon cancer cell lines (HCT-116 p53+/+ and p53−/−) and were inactive on FHs74Int normal human intestinal cell lines [13]. Alpha-terpineol showed significant cytotoxicity against Hep G2, a hepatocellular carcinomic human cell line; HeLa, an epithelioid carcinomic cell line; MOLT-4, a human lymphoblastic leukemia T cell line; K-562, a human chronic myelogenous leukemia cell line; and CTVR-1, an early B cell line from the bone marrow cells of a patient with acute myeloid leukemia [14]. Different officinal plants of Lebanon, among them* Satureja montana*, have shown cytotoxic activity against human erythroleukemic K562 cells. Its major constituent was the alpha-terpineol which demonstrated important cytotoxicity on the same cell line. Yet, this essential oil and alpha-terpineol induced erythroid differentiation of K562 cells [15]. Hassan and collaborators [16] suggested that alpha-terpineol inhibits the growth of tumor cells through a mechanism that involves inhibition of the NF-*κ*B pathway.

### 1.2. Alloocimene

Okamura and collaborators [17] evaluated the cytotoxicity of 12 monoterpenes. The acyclic monoterpene alloocimene showed significant cytotoxic activity; its 50% inhibitory concentration (IC_50_) was the highest of 12, for mouse P388 leukemia cell among others.

### 1.3. Menthol

Menthol was cytotoxic for murine leukemia WEHI-3 cells in a concentration-dependent manner. The* in vivo* activity on WEHI-3 cells was also examined [18]. In SNU-5 cells, menthol induced cytotoxicity by inhibiting the expression of topoisomerases I, II alpha, and II beta and promoting the expression of NF-*κ*B [19]. This compound also enhances the antiproliferative activity of 1*α*,25-dihydroxyvitamin D3 in LNCaP cells [20]. Wang and collaborators [21] showed that menthol inhibited the proliferation and motility of prostate cancer DU145 cells. Li and collaborators [22] demonstrated that menthol induced cell death via the TRPM8 channel in a human bladder cancer cell line. Okamoto and collaborators [23] also studied the role of menthol in the blockade of TRPM8 activity and found that it reduced the invasion potential of oral squamous carcinoma cell lines.

### 1.4. Beta-Dolabrin

Beta-dolabrin presented* in vitro* cytotoxic effects against Ehrlich's ascites carcinoma and KATO-III human stomach cancer cell line [24].

### 1.5. Alpha- and Gamma-Thujaplicin

Alpha-thujaplicin inhibited cell growth of Ehrlich's ascites carcinoma and KATO-III human stomach cancer [25]. Gama-thujaplicin also showed strong cytotoxic effects against KATO-III and Ehrlich's ascites carcinoma at 0.32 *μ*m/mL, with 85% and 91% inhibition of cell growth, respectively [24].

### 1.6. Borneol

The cytotoxicity of borneol and its DNA-damaging effects were studied in malignant HepG2 hepatoma cells, malignant Caco-2 colon cells, and nonmalignant human VH10 fibroblasts. Borneol showed cytotoxicity in all cell lines and did not cause DNA strand breaks at the concentrations studied. With respect to DNA-protective effects, borneol protected HepG2 and VH10 cells, but not Caco-2 cells, against H_2_O_2_-induced DNA damage [26]. Su and collaborators [27] demonstrated that borneol potentiates selenocystine-induced apoptosis in human hepatocellular carcinoma cells by enhancement of cellular uptake and activation of ROS-mediated DNA damage.

### 1.7. Ascaridole

Ascaridole exerts cytotoxic activity against different tumor cell lines (CCRF-CEM, HL60, and MDA-MB-231) [28]. Bezerra and collaborators [29] investigated the cytotoxicity and antitumor activity of ascaridole and in HL-60 and HCT-8 cells lines found IC_50_ values of 6.3 and 18.4 *μ*g/mL, respectively. Results from* in vivo* studies using sarcoma 180 as a tumor model demonstrated inhibition rates of 33.9% at 10 mg/kg and 33.3% at 20 mg/kg.

### 1.8. Carvacrol

Carvacrol produced significant cytotoxic activity against mouse leukemia P388 [30] and Hep-2 [31]. Horvathova and collaborators [32, 33] found that carvacrol exerted cytotoxic effects in K562, HepG2, and colonic Caco-2 cells and significantly reduced the level of DNA damage induced in these cells by the strong oxidant H_2_O_2_. The cytotoxic and DNA-protective effects of carvacrol were also demonstrated by Slamenova and collaborators [34]. Carvacrol displays cytotoxicity against B16-F10 melanoma cells and this cytotoxicity is reduced by the addition of vitamin C and vitamin E. In the P815 mastocytoma cell line, carvacrol showed a dose-dependent cytotoxic effect, but when tested on normal human peripheral blood mononuclear cells, it showed a proliferative effect rather than a cytotoxic one [31]. In the work of Stammati and collaborators [35], the authors compared the cytotoxic effects and molecular mechanisms of 5 monoterpenes: carvacrol, thymol, carveol, carvone, and isopulegol. Yin and collaborators [36] have proved the involvement of apoptosis in the cytotoxic effects of carvacrol on HepG2 cells. Arunasree [37] investigated the mechanism of carvacrol-induced cell death in MDA-MB 231 human metastatic breast cancer cells and demonstrated that this compound induced apoptosis in a dose-dependent manner [37]; the mechanism of action of carvacrol may in fact be related to its antioxidant activity and not associated with a DNA-damaging effect. Jayakumar and collaborators [38] demonstrated that carvacrol protects the antioxidant system in DEN-induced hepatocellular carcinogenesis. Carvacrol induced cell cycle arrest at S phase and induced apoptosis in P815 tumor cell line [39]. Zeytinoglu and collaborators [40] found that carvacrol inhibited growth of myoblast cells even after activation of a mutated N-ras oncogene. The essential oil of* Origanum onites* and carvacrol, its major constituent, showed strongly inhibition of the mutagenicity induced by 4-nitro-o-phenylenediamine and 2-aminofluorene using* Salmonella typhimurium* strains TA98 and TA100. These results indicate that the essential oil and carvacrol have pharmacological importance for the prevention of cancer because of its significant antimutagenicity effect [41]. The carcinogenesis-reducing potential of carvacrol was demonstrated by Ozkan and Erdogan [42]. Carvacrol was also tested against lung tumors induced by dimethylbenz[*α*]anthracene (DMBA) in rats* in vivo* and it was found to have strong antitumor activity at 0.1 mg/kg i.p. Although the mechanism of action of antitumor activity of carvacrol was not investigated in this study, evidence for an inhibitory effect on angiogenesis was observed [43].

### 1.9. *p*-Mentha-1,3,5-triene-2,3,6-triol

From the methanol extract of* Majorana syriaca*, Hirobe and collaborators [44] isolated the* p*-mentha-1,3,5-triene-2,3,6-triol. The screening for cytotoxicity on P388 cells showed significant activity for its monoterpene.

### 1.10. Terpinene

Terpinene showed significant evidence for antioxidant activity and cytotoxic activity against mouse leukemia P388 cells [31]. Ferraz and collaborators [9] evaluated the cytotoxicity of the essential oil of* Lippia gracilis* and its constituents against HepG2, K562, and B16-F10 tumor cell lines. Terpinene showed cytotoxic activity selectively for B16-F10 cells.

### 1.11. Thymol

Thymol presented cytotoxic effect against Hep-2 cells [35], P815 mastocytoma cells [31], HepG2 human hepatoma cells, Caco-2 human colonic cells, and V79 hamster lung cells [34]. Thymol showed antioxidant activity and cytotoxic activity against the mouse leukemia P388 cell line [30, 44]. Jayakumar and collaborators [38, 42] demonstrated that thymol is cytotoxic against HepG2 human hepatoma cells, colonic Caco-2 cells, and K562 cells, via a mechanism that may be related to antioxidant activity and not associated with a DNA-damaging effect. The effects of thymol on murine B16-F10 melanoma cells were tested by Paramasivam and collaborators [45], and thymol exhibited cytotoxicity with an IC_50_ value of 88.5 *μ*g/mL. Thymol cytotoxicity was reduced by addition of vitamin C and vitamin E. Radical scavengers (butylated hydroxytoluene and butylated hydroxyanisole) were able to significantly recover cell viability. Yin and collaborators [36] demonstrated that thymol induced cell cycle arrest at G0/G1 phase. Deb and collaborators [12] demonstrated that thymol induced apoptosis in HL-60 cells via caspase-dependent and caspase-independent pathways. Oskan and collaborators [42] demonstrated the antioxidant activity and carcinogenesis-reducing potential of thymol. In the work of Jaafari and collaborators [46], the authors compared the cytotoxic effects and molecular mechanisms of 5 monoterpenes: carvacrol, thymol, carveol, carvone, and isopulegol.

### 1.12. Thymohydroquinone

Studies have shown significant cytotoxic activity for thymohydroquinone in squamous cell carcinoma (SCC VII) and fibrosarcoma (FsaR) cell lines. This activity was dose dependent and more effective against tumor cells than L929 fibroblasts. Thymohydroquinone also showed a tumor inhibition rate of 52%* in vivo* [47]. On the other hand, Johnson and collaborators [48] showed that the reduction of thymoquinone to thymohydroquinone resulted in a 1.7-fold decrease in its cytotoxic potency against PC-3 tumor cells [48].

### 1.13. Thymoquinone

Thymoquinone possesses antiproliferative and proapoptotic activities in several cell lines [48–50]. Ivankovic and collaborators [47] showed cytotoxicity and also antitumor activity of thymoquinone. Cecarini and collaborators [51] demonstrated that thymoquinone induced time-dependent selective proteasome inhibition in glioblastoma cells and isolated enzymes and suggested that this mechanism could be implicated in the induction of apoptosis in cancer cells. The activity of thymoquinone against nonsmall cell lung cancer (NSCLC) and small cell lung cancer (SCLC) cell lines, alone and in combination with cisplatin (CDDP), was evaluated by Jafri and collaborators [52]. The authors observed that thymoquinone inhibited cell proliferation, reduced cell viability, and induced apoptosis. Thymoquinone inhibited cell proliferation by nearly 90% and showed synergistic effects with cisplatin. Thymoquinone was able to induce apoptosis in NCI-H460 and NCI-H146 cell lines. In a mouse xenograft model, the combination of thymoquinone and CDDP was well tolerated and significantly reduced tumor volume and tumor weight. Badary and collaborators [53] investigated the effects of thymoquinone on cisplatin-induced nephrotoxicity in mice and rats, and results revealed that thymoquinone induced amelioration of cisplatin nephrotoxicity and potentiated its antitumor activity. This natural product is also capable of improving the therapeutic efficacy of ifosfamide by decreasing ifosfamide-induced nephrotoxicity and improving its antitumor activity [54]. The chemosensitizing effect of thymoquinone on conventional chemotherapeutic agents was also demonstrated by Banerjee and collaborators.* In vitro* studies demonstrated that preexposure of cells to thymoquinone followed by gemcitabine or oxaliplatin resulted in greater growth inhibition compared with gemcitabine or oxaliplatin used alone. The mechanism involves downregulation of nuclear factor-*κ*B (NF-*κ*B), Bcl-2 family genes, and NF-*κ*B-dependent antiapoptotic genes [55]. Thymoquinone downregulated NF-*κ*B expression, which may explain its various cellular activities [52]. Sethi and collaborators [56] evaluated the involvement of suppression of the NF-*κ*B activation pathway in apoptosis induced by thymoquinone. Gali-Muhtasib and collaborators [57] demonstrated that thymoquinone triggered inactivation of the stress response pathway sensor CHEK1 and contributed to apoptosis in colorectal cancer cells. In human, multiple myeloma cells thymoquinone inhibited proliferation, induced apoptosis, and induced chemosensitization, through suppression of the signal transducer and activator of transcription 3 (STAT3) activation pathway [58]. Badary and collaborators [59, 60] demonstrated a powerful chemopreventive activity for thymoquinone against MC-induced fibrosarcoma tumors, suggesting that its mechanisms of action include antioxidant activity and interference with DNA synthesis, coupled with enhancement of detoxification processes [59, 60]. Barron and collaborators [61] examined the effects of thymoquinone and selenium (an endogenous antioxidant) on the proliferation of MG 63 osteoblasts cells in tissue culture and found that the combined use of these substances may be an effective treatment option against human osteosarcoma cells. The utilization of thymoquinone in the treatment of human osteosarcoma is also suggested by Roepke and collaborators [62], who showed that it induced p53-independent apoptosis, which is important because loss of p53 function is frequently observed in osteosarcoma patients. In contrast, Peng and collaborators [63] demonstrated antitumor and antiangiogenesis effects of thymoquinone on osteosarcoma through the NF-*κ*B pathway. Yazan and collaborators [64] reported that thymoquinone was cytotoxic to HeLa cells in a dose- and time-dependent manner and induced apoptosis via a p53-dependent pathway. Reactive oxygen species were also involved in mediating thymoquinone-induced apoptosis in a panel of human colon cancer cells (Caco-2, HCT-116, LoVo, DLD-1, and HT-29) through activation of ERK and JNK signaling [65]. Wilson-Simpson and collaborators [66] evaluated the participation of thymoquinone in the treatment of ES-2 ovarian tumor cells, and Farah and collaborators [67] evaluated the effects of antioxidants and thymoquinone on the cellular metabolism of A549 cells. Zubair and collaborators [68] demonstrated that redox cycling of endogenous copper by thymoquinone led to ROS-mediated DNA breakage and cell death. Talib and Abu Khader [69] studied the combinatorial effects of thymoquinone on the anticancer activity and hepatotoxicity of the prodrug CB 1954. Furthermore, findings from Richards and collaborators [70, 71] revealed that sustained delivery of antioxidants with thymoquinone may be a means of treating prostate cancer safely and effectively. In HEp-2 human laryngeal carcinoma cells, GSH depletion and caspase 3 activation mediated thymoquinone-induced apoptosis [49]. Caspase 3 activation (as well as caspase 8 and caspase 9) is related to thymoquinone-induced apoptosis in p53-null HL-60 cancer cells [72]. In prostate cancer cells, thymoquinone induced GSH depletion and increased ROS generation [73]. Shoieb and collaborators [74] demonstrated that the mechanism of action of thymoquinone on cancer cells involves apoptosis and cell cycle arrest. Apoptosis and cell cycle arrest were also evidenced in the studies of Hassan and collaborators [75] in the HepG2 hepatocellular carcinoma cell line and in the studies of Gali-Muhtasib and collaborators [76] in primary mouse keratinocytes, papilloma (SP-1), and spindle (I7) carcinoma cells. Gurung and collaborators [77] suggested that in glioblastoma cells thymoquinone induced DNA damage, telomere attrition through telomerase inhibition, and cell death. More recently, Paramasivam and collaborators [78] showed that thymoquinone produced cytotoxic effects on Neuro-2a mouse neuroblastoma cells through caspase 3 activation, with downregulation of XIAP. Abusnina and collaborators [79] demonstrated that thymoquinone induces acute lymphoblastic leukemia cell apoptosis. Thymoquinone also has potential as a novel therapeutic agent against pancreatic cancer. Torres and collaborators [80] demonstrated that thymoquinone downregulated MUC4 expression in pancreatic cancer cells and induced apoptosis by two different pathways. The activity of thymoquinone against multidrug resistant (MDR) human tumor cell lines was also evaluated by Worthen and collaborators [81]. el-SA and collaborators [82] show that thymoquinone upregulated PTEN expression and induced apoptosis in doxorubicin-resistant human breast cancer cells. This study suggested that thymoquinone may not be an MDR substrate and that radical generation may not be critical to its cytotoxic activity [81]. Ravindram and collaborators [83] demonstrated that encapsulation of thymoquinone into nanoparticles enhanced its antiproliferative and chemosensitizing effects. The same type of study was conducted by Ganea and collaborators [84]. Wirries and collaborators [85] reported that structural modifications may contribute to the further clinical studies with thymoquinone. Banerjee and collaborators [86] and Effenberger and collaborators [87] also analyzed thymoquinone analogs with potential cytotoxicity against cancer cells. El-Najjar and collaborators [88] showed that bovine serum albumin played a protective role against thymoquinone-induced cell death. Al-Shabanah and collaborators [89] demonstrated that thymoquinone protected against doxorubicin-induced cardiotoxicity without compromising its antitumor activity. Nagi and Almakki [90] investigated a potential role for thymoquinone in protection against chemical carcinogenesis and toxicity by inducing quinone reductase and glutathione transferase in mice liver. Thymoquinone inhibited proliferation, induced apoptosis, and chemosensitized human multiple myeloma cells through suppression of the signal transducer and activator of transcription 3 (STAT3) activation pathway [91]. Rajput and collaborators [92] showed that molecular targeting of Akt by thymoquinone promoted G1 arrest through translation inhibition of Cyclin D1 and induced apoptosis in breast cancer cells. Effenberger-Neidnicht and collaborators [93] showed that thymoquinone boosted the anticancer effects of doxorubicin in certain cancer cell. Tundis and collaborators [94] demonstrated the possible involvement of the PPAR-*γ* pathway in the anticancer activity of thymoquinone in breast cancer cells. Thymoquinone enhances survival and activity of antigen-specific CD8-positive T cells* in vitro*, a result that can be useful in the cancer therapy [95]. Exposure of cancer cells derived from lung, liver, colon, melanoma, and breast to increasing thymoquinone concentrations presented a significant inhibition of viability with an inhibition of Akt phosphorylation, DNA damage, and activation of mitochondrial proapoptotic pathways. Thymoquinone inhibited the invasive potential of various cancer cells. Moreover, thymoquinone synergizes with cisplatin to inhibit cellular viability. Tumor growth inhibition was associated with a significant increase in activated caspase 3.* In silico* target identification suggested several potential targets of thymoquinone, in particular HDAC2 proteins and 15-hydroxyprostaglandin dehydrogenase [96]. Lang and collaborators [97] showed that thymoquinone interfered with polyp progression in ApcMin mice through induction of tumor-cell specific apoptosis and modulation of Wnt signaling through activation of GSK-3*β*. Thymoquinone also induced apoptosis in oral cancer cells through P38*β* inhibition [98]. Odeh and collaborators [99] described the encapsulation of thymoquinone into a liposome, which maintained stability and improved bioavailability, while it maintained anticancer activity. Das and collaborators [100] showed that thymoquinone and diosgenin, alone and in combination, inhibited cell proliferation and induced apoptosis in squamous cell carcinoma. Alhosin and collaborators [101] demonstrated that thymoquinone induced degradation of *α*- and *β*-tubulin proteins in human cancer cells without affecting their levels in normal human fibroblasts.

### 1.14. Myrcene

Myrcene showed significant cytotoxic effects in crown gall tumors, MCF-7 breast carcinoma, HT-29 colon adenocarcinoma [102], and other cell lines [9]. Silva and collaborators [103] investigated the cytotoxicity of myrcene against HeLa (human cervical carcinoma), A-549 (human lung carcinoma), HT-29 (human colon adenocarcinoma), and Vero (monkey kidney) cell lines as well as mouse macrophages. Okamura and collaborators [17] performed a screening of 12 monoterpenes. Among them, the acyclic monoterpene, myrcene, exhibited significant cytotoxicity against P388 leukemia cell.

### 1.15. Sobrerol

Sobrerol showed anticarcinogenic activity during the initiation phase of DMBA-induced carcinogenesis, which was mediated through induction of the hepatic detoxification enzymes glutathione-S-transferase and uridine diphosphoglucuronosyl transferase [104].

### 1.16. Limonene

Studies have demonstrated the antitumorigenic effects of limonene against pancreatic cancer and breast cancer [105]. Limonene showed antioxidant and radical scavenging activities in several model systems and cytotoxicity against MCF-7, K562, PC 12 [106], A-549, HT-29 cell lines [107], and HepG2 hepatocarcinoma cell lines [108]. Bhattacharjee and Chatterjee [109] promoted the identification of proapoptotic, anti-inflammatory, antiproliferative, anti-invasive, and potential antiangiogenic activities of limonene by employing a dual reverse virtual screening protocol. A probabilistic set of antitumor targets was generated, which can be further confirmed by* in vivo* and* in vitro* experiments. Ji and collaborators [110] demonstrated that induction of apoptosis by d-limonene was mediated by a caspase-dependent mitochondrial death pathway in human leukemia cells. Furthermore, d-limonene induced apoptosis in HL-60 cells through activation of caspase-8 [111]. Pattanayak and collaborators [112] verified that limonene inhibited the activity of HMG-CoA reductase due to greater binding affinity with the receptor and thus reduced the possibility of cancer growth. Chen and collaborators [113] suggested that the anticancer activity of limonene might be related to inhibition of the membrane association of P21ras protein and increased gap junction intercellular communication. Haag and collaborators [114] demonstrated that limonene induced regression of mammary carcinomas, and when given in combination with 4-hydroxyandrostrenedione it resulted in greater rat mammary tumor regression (83.3%) than either agent given alone [115]. Elson and collaborators [116] demonstrated that limonene reduced the average number of rat mammary carcinomas that developed in 7,12-dimethylbenz[*α*]anthracene-treated rats when the terpene was fed during the initiation or promotion/progression stages of carcinogenesis. Chidambara and collaborators [117] tested citrus volatile oil rich in d-limonene and verified that the oil induced apoptosis and acted as an antiangiogenic with a preventative effect on colon cancer. Limonene also showed a selective antiproliferative action on tumor lymphocytes [118], and it inhibited the metastatic progression of B16F-10 melanoma cells in mice [119]. Limonene had anticarcinogenic activity when fed during the initiation stage of DMBA-induced rat mammary carcinogenesis, and this mechanism was mediated through the induction of the hepatic detoxification enzymes glutathione-S-transferase and uridine diphosphoglucuronosyl transferase [104]. Gelb and collaborators [107] tested the ability of limonene to inhibit protein prenylation enzymes* in vitro* and found that it was a weak inhibitor of both mammalian and yeast protein farnesyltransferase (PFT) as well as protein geranylgeranyl transferase (PGGT). D-Limonene is an effective inhibitor of 4-(methylnitrosamino)-1-(3-pyridyl)-1-butanone metabolic activation [120]. Elegbede and Gould [121] investigated the effects of limonene at the initiation stage of aflatoxin B1-induced hepatocarcinogenesis and found that limonene significantly inhibited aflatoxin-DNA adduct formation in hepatocytes, which suggested that limonene may have potential as a chemopreventive agent against aflatoxin-induced liver cancer. D-Limonene inhibited the development of colonic aberrant crypt foci induced by azoxymethane in F344 rats, which suggested that this monoterpenoid might be a chemopreventive agent for colonic carcinogenesis in rats [111]. D-Limonene induced GST activity 2.4–3.0-fold higher than controls in the mouse liver and mucosa of the small intestine and large intestine, which suggested chemopreventive activity [122]. Parija and Ranjan [123] demonstrated the involvement of YY1 in NDEA-induced hepatocarcinogenesis and chemoprevention mediated by d-limonene.

### 1.17. *p*-Mentha-2,8-dien-1-ol and* p*-Mentha-8(9)-en-1,2-diol

Zheng and collaborators [124] demonstrated the ability of* p*-mentha-2,8-dien-1-ol and* p*-mentha-8(9)-en-1,2-diol to inhibit benzo[*α*]pyrene-induced carcinogenesis in the mouse forestomach. The number of tumors per mouse was also significantly decreased by these compounds. No tumor inhibition was observed with* p*-mentha-2,8-dien-1-ol.

### 1.18. Perillic Acid

Yeruva and collaborators [125] demonstrated that perillic acid demonstrated dose-dependent cytotoxicity in A549 and H520 cell lines, inducing cell cycle arrest and apoptosis. Combination studies revealed that previous exposure of cells to perillic acid sensitized the cells to cisplatin and radiation in a dose-dependent manner. Samaila and collaborators [126] showed that perillic acid has potential for use as a radiosensitizer in chemoradiation therapy of head and neck cancers.

### 1.19. Perillyl Alcohol

Stark and collaborators [127] and Burke and collaborators [128] demonstrated that perillyl alcohol has antitumor activity against pancreatic carcinomas at nontoxic doses and may be an effective chemotherapeutic agent for human pancreatic cancer. The antitumor activity of perillyl alcohol against pancreatic cancers may stem from its ability to inhibit the prenylation of growth-regulatory proteins other than K-Ras, including H-Ras [129]. Furthermore, the antitumor activity of perillyl alcohol in pancreatic cancers may be due to preferential stimulation of Bak-induced apoptosis in malignant cells compared to normal cells [130]. Further studies to evaluate the cytotoxicity mechanisms of perillyl alcohol against pancreatic cancer cells were conducted by Lebedeva and collaborators [131]. Sundin and collaborators [132] demonstrated that the perillyl alcohol inhibited telomerase activity in prostate cancer cells. Perillyl alcohol in combination with STI571 enhances the ability of STI571 to inhibit proliferation and induce apoptosis in K562 cells [133]. In bcr/abl-transformed leukemia cells perillyl alcohol induced c-myc-dependent apoptosis [134]. In A549 and H520 cell lines, Yeruva and collaborators [125] demonstrated that perillyl alcohol presented dose-dependent cytotoxicity with cell cycle arrest and apoptosis. Elevated expression of bax, p21, and increased caspase 3 activity were evidenced. Other studies revealed that perillyl alcohol sensitized cancer cells to cisplatin and radiation in a dose-dependent manner. Perillyl alcohol attenuated* in vitro* angiogenesis, modulated angiogenic factor production, and inhibited cell proliferation and survival in endothelial and tumor cells [135]. Loutrati and collaborators [136] also demonstrated that perillyl alcohol, in additional to its anticancer activity, may be an effective agent in the treatment of angiogenesis-dependent diseases. Garcia and collaborators [137] demonstrated that perillyl alcohol is an Na/K-ATPase inhibitor and suggested that its antitumor action could be linked to its Na/K-ATPase binding properties. Perillyl alcohol reduced 21–26 kDa proteins isoprenylation to 50% of the control level at a concentration of 1 mM but had no effect on the isoprenylation of 67, 47, or 17 kDa proteins [138]. Sahin and collaborators [139] demonstrated that perillyl alcohol selectively induced G0/G1 arrest and apoptosis in Bcr/Abl-transformed myeloid cell lines. In the same year Satomi and collaborators [140] demonstrated induction of AP-1 activity by perillyl alcohol in breast cancer cells. Ahn and collaborators [141] verified that cytotoxicity of perillyl alcohol against cancer cells is potentiated by hyperthermia. Ren and Gould [142] demonstrated an inhibition of ubiquinone and cholesterol synthesis by perillyl alcohol, and the authors suggested that these effects may contribute to the antitumor activity of the molecule. Manassero and collaborators [108] tested the ability of perillyl alcohol to inhibit protein prenylation enzymes* in vitro* and verified that it is a weak inhibitor of both mammalian and yeast forms of protein farnesyltransferase and protein geranylgeranyl transferase. In NIH3T3 cells, Ren and collaborators [143] verified that perillyl alcohol inhibited the* in vivo* prenylation of specific proteins by type I and type II geranylgeranyl-protein transferases but not by farnesyl-protein transferase. Elegbede and Gould [121] investigated the effects of perillyl alcohol at the initiation stage of aflatoxin B1-induced hepatocarcinogenesis. In this study, analysis of liver samples showed that perillyl alcohol significantly inhibited aflatoxin-DNA adduct formation in hepatocytes, and therefore this monoterpene may have potential for use as a chemopreventive agent against aflatoxin-induced liver cancer. Balassiano and collaborators [144] observed the effects of perillyl alcohol in the glial C6 cell line* in vitro* and antimetastatic activity in a chorioallantoic membrane model and suggested a possible use for perillyl alcohol as an* in vivo* antimetastatic drug. Da Fonseca and collaborators [145] discussed perillyl alcohol intranasal delivery as a potential antitumor agent. The chemopreventive effect of topical application of perillyl alcohol on DMBA-initiated and 12-O-tetradecanoylphorbol-13-acetate- (TPA-) promoted skin tumorigenesis and its mechanisms of action were investigated in Swiss albino mice [146]. The mechanisms of action of perillyl alcohol were investigated in advanced rat mammary carcinomas by Ariazi and collaborators [147], and it was found that it activated the TGF-beta signaling pathway and induced cytostasis and apoptosis in mammary carcinomas. These authors also identified differentially expressed genes in mammary carcinomas treated with perillyl alcohol. Perillyl alcohol-mediated cell cycle arrest was found to precede apoptosis, which raised the possibility that the primary effect of perillyl alcohol is to induce G0/G1 arrest, with apoptosis as a consequence of this growth arrest [139, 148]. Using a novel and innovative approach, Lebedeva and collaborators [149] demonstrated that chemoprevention by perillyl alcohol, coupled with viral gene therapy, reduced pancreatic cancer pathogenesis. Perilla aldehyde is a major intermediary metabolite of perillyl alcohol in the rat* in vivo* and may contribute to the anticancer effect of perillyl alcohol [150]. Phillips and collaborators [151] investigated the pharmacokinetics of active drug metabolites after oral administration of perillyl alcohol in dogs. Samaila and collaborators [126] verified that perillyl alcohol has potential as a radiosensitizer in chemoradiation therapy of head and neck cancers. Rajesh and collaborators [152] also studied the role of perillyl alcohol as a radiosensitizer and chemosensitizer in malignant glioma. Ripple and collaborators [153] conducted a phase I dose-escalation trial of perillyl alcohol given p.o. on a continuous basis 4 times per day to characterize its maximum tolerated dose, toxicities, pharmacokinetic profile, and antitumor activity. This study was conducted after a phase I clinical trial of perillyl alcohol in which no objective tumor responses were noted when it was administered daily [154]. A phase I trial of perillyl alcohol in patients with advanced solid tumors was conducted by Azzoli and collaborators [155]. A phase I pharmacokinetic trial of perillyl alcohol in patients with refractory solid malignancies was performed by Hudes and collaborators [156], in which the authors verified that perillyl alcohol at 1600–2100 mg/m^2^ p.o. 3 times daily was well tolerated on a 14-day on/14-day off dosing schedule. A phase II trial of perillyl alcohol in patients with metastatic colorectal cancer was conducted by Meadows and collaborators [157], in which the authors found that oral perillyl alcohol did not have clinical antitumor activity when used for patients with advanced colorectal carcinoma, despite preclinical evidence of anticancer activity.

### 1.20. 1,8-Cineole/Eucalyptol

The cytotoxicity of 1,8-cineole was investigated against SK-OV-3, HO-8910, and Bel-7402 cell lines [158]. Monoterpene 1,8-cineole demonstrated moderate cytotoxicity in Hep G2, HeLa, MOLT-4, K-562, and CTVR-1 cell lines [14]. Asanova and collaborators [159] demonstrated that 1,8-cineole had moderate antioxidant and cytotoxic properties and pronounced analgesic and antitumor activity. Cha and collaborators [160] found that 1,8-cineole induced apoptosis in KB cells via mitochondrial stress and caspase activation. Bhattacharjee and Chatterjee [109] promoted the identification of proapoptotic, anti-inflammatory, antiproliferative, anti-invasive, and potential antiangiogenic activities of eucalyptol by employing a dual reverse virtual screening protocol. A probabilistic set of antitumor targets was generated, which can be further confirmed by* in vivo* and* in vitro* experiments.

### 1.21. Perilla Aldehyde

Perilla aldehyde showed marked antioxidant and radical scavenging activity using different model systems, including 1,1-diphenyl-2-picrylhydrazyl radical (DPPH) and beta-carotene-linoleic acid blenching assays, and also inhibited MCF-7, K562, and PC-12 cell growth in a dose- and time-dependent manner, with IC_50_ values that ranged from 0.25–5.0 mmol/L [106].

### 1.22. Terpinen-4-ol

Terpinen-4-ol showed cytotoxicity against Hep G2, HeLa, MOLT-4, K-562, CTVR-1 [14], and human M14 melanoma cells [161]. Bozzuto and collaborators [162] demonstrated that this monoterpene interfered with the migration and invasion processes of drug-sensitive and drug-resistant melanoma cells. Terpinen-4-ol also induced necrosis and cell cycle arrest in murine cancer cell lines [163].

### 1.23. Citral

Citral is cytotoxic against P388 mouse leukemia [164], HeLa [165], Ishikawa, and ECC-1 cancer cells [166]. Xia and collaborators [167] reported that citral had a therapeutic effect on leukemia.

### 1.24. Carvone

Carvone inhibited viability and proliferation of Hep-2 cells in a dose-dependent manner, with morphological analysis suggesting an involvement of apoptosis. In the SOS chromotest, carvone did not cause DNA damage at nontoxic doses. In the DNA repair test, a marked dose-dependent differential toxicity was observed [35]. Carvone also presented a dose-dependent cytotoxic effect against HeLa cells [168]. In contrast, more recently, Aydin and collaborators [169] reported that carvone could be a promising anticancer agent to improve brain tumor therapy. In the work of Jaafari and collaborators [46], the authors compared the cytotoxic effects and molecular mechanisms of five monoterpenes: carvacrol, thymol, carveol, carvone, and isopulegol. The results showed that the carvacrol is the most active monoterpene. However, the data of IC_50_ (0.17 *μ*M on K562 cells) showed that carvone has significant cytotoxicity. Although carvacrol induce cell cycle arrest in S phase, no effect on cell cycle was observed for carvone.

### 1.25. Alpha- and Beta-Pinene

Alpha- and beta-pinene showed cytotoxicity on tumor lymphocytes [106] and in others different tumor and nontumor cell lines [157, 170]. In the same cases, this cytotoxicity was comparable to doxorubicin [171]. Alpha- and beta-pinene did not show antitumor activity* in vivo* using the Ehrlich ascites tumor model [157]. The cytotoxic potential of alpha-pinene was investigated in SK-OV-3, HO-8910, Bel-7402 [158], and U937 cell lines [172]. The cytotoxicity of alpha-pinene was comparable to doxorubicin [173]. Bhattacharjee and Chatterjee [109] promoted the identification of proapoptotic, anti-inflammatory, antiproliferative, anti-invasive, and potential antiangiogenic activities of alpha-pinene by employing a dual reverse virtual screening protocol. Jin and collaborators [174] demonstrated that alpha-pinene triggered oxidative stress and related signaling pathways in A549 and HepG2 cells. The cytotoxic potential of beta-pinene was investigated in MCF-7, A375, and HepG2 cancer cells [94] and in other different tumor and nontumor cell lines [50].

### 1.26. Geraniol

Carnesecchi and collaborators [175] demonstrated that this monoterpene sensitized human colonic cancer cells to 5-Fluorouracil treatment* in vitro*. In a later work, Carnesecchi and collaborators [176] demonstrated that geraniol modulated DNA synthesis and potentiated 5-fluorouracil effects on human colon tumor xenografts. Bhattacharjee and Chatterjee [109] promoted the identification of proapoptotic, anti-inflammatory, antiproliferative, anti-invasive, and potential antiangiogenic activities of geraniol by employing a dual reverse virtual screening protocol. A probabilistic set of antitumor targets was generated, which can be further confirmed by* in vivo* and* in vitro* experiments. Geraniol suppressed pancreatic tumor growth without significantly affecting blood cholesterol levels [128]. Polo and de Bravo [177] demonstrated multiple effects of geraniol on mevalonate and lipid metabolism in Hep G2 cells that affected cell proliferation. More recently, Crespo and collaborators [178] reported transcriptional and posttranscriptional inhibition of HMGCR and PC biosynthesis by geraniol in 2 Hep-G2 cell proliferation-linked pathways. Geraniol inhibited the activity of HMG-CoA reductase, which reduced the possibility of cancer growth [112]. Geraniol also inhibited growth and polyamine biosynthesis in human colon cancer cells [179]. Zheng and collaborators [122] suggested that geraniol showed promise as a chemopreventive agent because it showed strong GST-inducing activity in the mucosa of the small intestine and the large intestine. Ong and collaborators [180] and Cardozo and collaborators [181] suggested that geraniol showed promising chemopreventive effects against hepatocarcinogenesis. More recently, Madankumar and collaborators [182] evidenced that geraniol presents chemopreventive potential against oral carcinogenesis.

### 1.27. Citronellol

Citronellol exhibited weak cytotoxic effects against HL60 tumor cells [183].

### 1.28. Camphene

Wright and collaborators [165] verified that the cytotoxic activity of humulene on MCF-7 cells was antagonized by camphene.

### 1.29. Linalool

Linalool showed cytotoxic effects on C32 cells [94], BCC-1/KMC, AGS, RTCC-1/KMC, U_2_OS, HeLa, H520, H661, OSCC-1/KMC, J82 [184], human leukemia, and lymphoma cell lines [185], amelanotic melanoma C32 cells, and renal cell adenocarcinoma cells [186]. Usta and collaborators [187] verified that linalool decreased HepG2 viability by inhibiting mitochondrial complexes I and II, increasing reactive oxygen species, and decreasing ATP and GSH levels. Gu and collaborators [188] showed that linalool preferentially induced robust apoptosis of a variety of leukemia cells by upregulation of p53 and cyclin-dependent kinase inhibitors. A study conducted by Ravizza and collaborators [189] demonstrated that linalool reversed doxorubicin resistance in human breast adenocarcinoma cells. Maeda and collaborators [190] demonstrated that linalool significantly suppressed HL60 cell proliferation, induced apoptosis, and promoted cell differentiation. The effect of linalool on doxorubicin-induced antitumor activity was evaluated by Miyashita and Sadzuca [191].

### 1.30. Cymene

The anticancer activity of* p*-cymene was studied by Bourgou and collaborators [50]. Ferraz and collaborators [9] investigated the cytotoxic effect of* p*-cymene in three cell lines: HepG2, K562, and B16-F10. The results demonstrated that* p*-cymene was cytotoxic only to B16-F10 cell lines, showing IC_50_ = 20.06 *μ*g/mL.

### 1.31. Terpinen-4-ol and *α*-Terpinolene

Badary [54] investigated the cytotoxicity of terpinen-4-ol against two different cell lines, A-549 and DLD-1. For both cell lines, this monoterpene induced weak cytotoxicity. In another study, the cytotoxicity of the* Helichrysum gymnocephalum* essential oil was evaluated [192]. In addition, this work aimed to establish correlations between the identified compounds and their biological activities (antiplasmodial and cytotoxic). They reviewed researches for essential oils having an activity against* P. falciparum* and/or on MCF-7 cell line in order to identify, by correlation, the main active compounds. The *α*-terpinolene, present in the oil, showed a higher relationship with the cytotoxic activity against MCF-7 cell [192].

## 2. Conclusions

Several studies have shown* in vitro* and* in vivo* antitumor activity of many essential oils obtained from plants. The antitumor activity of essential oils of many species has been related to the presence of monoterpenes in their composition [8–12]. This review shows that many monoterpenes are being examined for* in vitro* and* in vivo* antitumor activity, presenting important results that provide insights for the use of such compounds for the treatment of cancer. In addition, some studies show that monoterpenes are already in clinical phase trials for drug development, as in the case of Perillyl alcohol [145]. However, despite many studies showing the evaluation of possible mechanisms of action of these compounds, the majority of studies still present only preliminary screening data and therefore do not describe any mechanism of action. For these studies, the monoterpenes are classified only as “active.” Thus, additional research is needed to be developed to elucidate how various monoterpenes act to inhibit the proliferation and to induce tumor cell death.

## Figures and Tables

**Table 1:** Essential oils monoterpenes with antitumor activity.

Compound	Antitumor activity and/or mechanism	Animal/cell line tested	IC_50_, % survival, % mortality or % growth inhibition, or dose	Reference
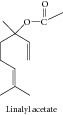	Active (cell cycle arrest; induction of apoptosis)	Human colon cancer cell lines HCT-116 (p53+/+)	10–30%^∗,a^	[13]
		Human colon cancer cell lines HCT-116 (p53−/−)	10–30%^∗,a^	

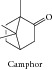	Active (cell cycle arrest; induction of apoptosis)	Human colon cancer cell lines HCT-116 (p53+/+)	10–30%^∗,a^	[13]
		Human colon cancer cell lines HCT-116 (p53−/−)	10–30%^∗,a^	

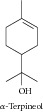		HepG2 (hepatocellular carcinomic human cell line)	ND	
		HeLa (epithelioid carcinomic cell line)	ND	
	Active (ND)	MOLT-4 (human lymphoblastic leukemia T-cell line)	ND	[14]
		K-562 (human chronic myelogenous leukemia cell line)	ND	
		CTVR-1 (early B-cell line from the bone marrow cells of a patient with acute myeloid leukemia)	ND	

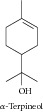	Active (ND)	K-562 (human chronic myelogenous leukemia cell line)	56.15 *µ*g/mL	[15]
	Active (inhibition of the NF-*κ*B pathway)	Small cell lung carcinoma	0.26 mM	[16]

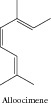	Active (ND)	Mouse P388 leukemia cell	34–54 *µ*g/mL∗	[17]

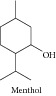	Active (decrease of Mac-3 and CD11b markers of macrophages and granulocytes precursors)	Murine leukemia WEHI-3 cells (*in vitro*) Murine leukemia WEHI-3 cells (*in vivo*)	ND 1 or 10 mg/kg	[18]
	Active (inhibition gene expression of topoisomerases I, II alpha, and II beta and promoting the gene expression of NF-*κ*B)	SNU-5 (human gastric carcinoma cell line)	1.62 mg/mL	[19]

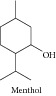	Active (combined with 1*α*,25-dihydroxyvitamin D3)	LNCaP (human prostate carcinoma cell line)	ND	[20]
	Active (TRPM8 channel activation; cell cycle arrest)	DU145 (human prostate carcinoma cell line)	53.41–90.66%^∗,a^	[21]
	Active (mitochondrial membrane depolarization via the TRPM8 channel)	T24 (Human bladder cancer cell line)	ND	[22]
	Active (agonist of TRPM8)	Oral squamous carcinoma cell lines (HSC3 and HSC4)	ND	[23]

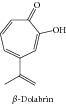	Active (ND)	KATO-III (human stomach cancer cell line)	67%^b^	[24]
		Ehrlich's ascites carcinoma	75%^b^	

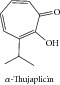	Active (ND)	KATO-III (human stomach cancer cell line)	86%^b^	[25]
		Ehrlich's ascites carcinoma	87%^b^	

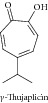	Active (ND)	KATO-III (human stomach cancer cell line)	85%^b^	[24]
		Ehrlich's ascites carcinoma	91%^b^	

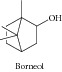	Active (ND)	HepG2 (hepatocellular carcinomic human cell line)	2750 *µ*M	[26]
		Caco-2 (colon malignant cell line)	2250 *µ*M	
	Active (potentiates selenocystine-induced apoptosis and activation of ROS-mediated DNA damage)	HepG2 (hepatocellular carcinomic human cell line)	ND	[27]

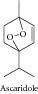		CCRF-CEM (human T cell lymphoblast-like cell line)	ND	
	Active (ND)	HL60 (acute promyelocytic cancer cell line)	ND	[28]
		MDA-MB-231 (Human metastatic breast cancer cell line)	ND	
	Active (ND)	HL-60 (acute promyelocytic cancer cell line)	6.3 *µ*g/mL	[29]
		HCT-8 (ileocecal colorectal adenocarcinoma)	18.4 *µ*g/mL	

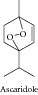	Active (ND)	Sarcoma 180 (*in vivo*)	10 or 20 mg/kg	[29]

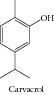	Active (ND)	Mouse leukemia P388 cell line	ND	[30]
		P815 (mastocytoma cell line)	<0.004% v/v	[31]
		P815 (mastocytoma cell line)	1.2% v/v *·* 10^−2^	[39]
		K-562 (human chronic myelogenous leukemia)	1.2% v/v *·* 10^−2^	[39]
	Active (cell cycle arrest; induction of apoptosis)	CEM (acute T lymphoblastic leukemia)	1.2% v/v *·* 10^−2^	[39]
		MCF-7 (human breast adenocarcinoma)	2.5% v/v *·* 10^−2^	[39]
		MCF-7 gem (human breast adenocarcinoma resistant to gemcitabine)	0.85% v/v *·* 10^−2^	[39]
		P815 (mastocytoma cell line)	0.067 *µ*M	[46]
		K-562 (human chronic myelogenous leukemia)	0.067 *µ*M	[46]

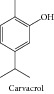		CEM (acute T lymphoblastic leukemia)	0.042 *µ*M	[46]
	Active (cell cycle arrest; induction of apoptosis)	MCF-7 (human breast adenocarcinoma)	0.125 *µ*M	[46]
		MCF-7 gem (human breast adenocarcinoma resistant to gemcitabine)	0.067 *µ*M	[46]
	Active (induction of apoptosis)	Hep2 (larynx epidermoid carcinoma)	0.22–0.32 mM∗	[35]
	Active (induction of apoptosis)	HepG2 (hepatocellular carcinomic human cell line)	0.4 mmol/L	[36]
	Active (induction of apoptosis)	MDA-MB 231 (human metastatic breast cancer cell line)	100 *μ*M	[37]
		HepG2 (hepatocellular carcinomic human cell line)	ND	[32]
	Active (antioxidant activity)	Caco-2 (colon malignant cell line)	ND	[32]
		K562 (erythromyeloblastoid leukemia cell line)	150–200 *μ*M	[33]
	Active (ND)	HepG2 (hepatocellular carcinomic human cell line)	350 *μ*M	[34]
		Caco-2 (colon malignant cell line)	600 *μ*M	

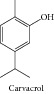	Active (Inhibition of DNA synthesis)	Lung tumors induced by ^1^DMBA in rats	0.1 mg/kg	[43]
		Myoblast cells	60 *μ*g/mL	[40]
	Active (prevention of hepatocellular carcinogenesis)	DEN-induced hepatocellular carcinogenesis	15 mg/kg	[38]
	Active (ND)	HepG2 (hepatocellular carcinomic human cell line)	53.09 *μ*g/mL	[42]

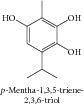	Active (ND)	Mouse leukemia P388 cell line	1.1 *µ*g/mL	[44]

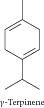	Active (ND)	Mouse leukemia P388 cell line	ND	[30]
		HepG2 (hepatocellular carcinomic human)	>25 *µ*g/mL	
	Active (cell cycle arrest; induction of apoptosis)	K562 (erythromyeloblastoid leukemia cell line)	ND	[9]
		B16-F10 (melanoma)	9.28 *µ*g/mL	

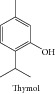	Active (ND)	Hep-2 (larynx epidermoid carcinoma)	0.71–0.78 mM∗	[35]
	Active (ND)	HepG2 (hepatocellular carcinomic human cell line)	400 *μ*M	[34]
		Caco-2 (colon malignant cell line)	700 *μ*M	
	Active (ND)	Mouse leukemia P388 cell line	ND	[30]
	Active (ND)	Mouse leukemia P388 cell line	0.80 *µ*g/mL	[44]
		P815 (mastocytoma cell line)	0.015% v/v	[31]
		P815 (mastocytoma cell line)	3.1% v/v *·* 10^−2^	[39]
		K-562 (human chronic myelogenous leukemia)	>22% v/v *·* 10^−2^	[39]
	Active (cell cycle arrest and apoptosis)	CEM (acute T lymphoblastic leukemia)	6.9% v/v *·* 10^−2^	[39]
		MCF-7 (human breast adenocarcinoma)	>22% v/v *·* 10^−2^	[39]
		MCF-7 gem (human breast adenocarcinoma resistant to gemcitabine)	>22% v/v *·* 10^−2^	[39]
		P815 (mastocytoma cell line)	0.15 *µ*M	[46]
		K-562 (human chronic myelogenous leukemia)	0.44 *µ*M	[46]

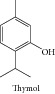		CEM (acute T lymphoblastic leukemia)	0.31 *µ*M	[46]
	Active (cell cycle arrest and apoptosis)	MCF-7 (human breast adenocarcinoma)	0.48 *µ*M	[46]
		MCF-7 gem (human breast adenocarcinoma resistant to gemcitabine)	ND	[46]
		HepG2 (hepatocellular carcinomic human cell line)	ND	[32]
	Active (antioxidant activity)	Caco-2 (colon malignant cell line)	ND	[32]
		K562 (erythromyeloblastoid leukemia cell line)	400–500 *µ*M	[33]
	Active (cell cycle arrest; induction of apoptosis)	HL-60 (acute promyelocytic cancer cell line)	ND	[12]
	Active (ND)	HepG2 (hepatocellular carcinomic human cell line)	60.01 *µ*g/mL	[42]

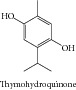		SCC VII (squamous cell carcinoma)	87%^c^	
	Active (ND)	Fibrosarcoma (FsaR) cell lines (*in vitro*)	92%^c^	[47]
		Fibrosarcoma (FsaR) cell lines (*in vivo*)	20 mg/kg	

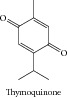		A549 (lung carcinoma cell line)	72.0–146 *µ*M∗	
	Active (induction of apoptosis)	HEp-2 (larynx epidermoid carcinoma cell line)	22.9–34.6 *µ*M^∗ ^	[49]
		HT-29 (colon adenocarcinoma cell line)	51.0–53.3 *µ*M∗	
		MIA PaCa-2 (pancreas carcinoma cell line)	60.0–67.9 *µ*M∗	
		SF-539 (central nervous system cancer cell line)	ND	
		PC-3 (prostate cancer cell line)	ND	
	Active (ND)	M-14 (melanoma)	ND	[48]
		OVCAR-5 (ovarian cancer cell line)	ND	
		MCF-7 (breast adenocarcinoma cell line)	ND	
	Active (ND)	A-549 (lung carcinoma cell line)	13.0 *µ*M	[50]
		DLD-1 (colorectal adenocarcinoma cell line)	5.9 *µ*M	
		SCC VII (squamous cell carcinoma)	86%^c^	
	Active (ND)	Fibrosarcoma (FsaR) cell lines (*in vitro*)	92%^c^	[47]
		Fibrosarcoma (FsaR) cell lines (*in vivo*)	20 mg/kg	

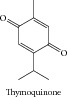	Active (proteasome inhibition and induction of apoptosis)	U87 MG (malignant glioma cells) T98G (malignant glioma cells)	61.46–77.73 *µ*M∗ 35.83–47.08 *µ*M∗	[51]
	Active (induction of apoptosis)	NCI-H460 (nonsmall cell lung cancer cell line) NCI-H146 (small cell lung cancer cell line)	ND ND	[52]
	Active (downregulation of NF-*κ*B expression)	Mouse xenograft model using NCI-H460 (human large cell lung cancer)	20 mg/kg	[52]
	Active (suppression of the NF-*κ*B activation pathway and induction of apoptosis)	KBM-5 (human myeloid cell line) A293 (human embryonic kidney cell line)	ND ND	[56]
	Active (suppression of STAT3 activation and induction of apoptosis)	Multiple myeloma	ND	[58]
	Active (inactivation of the stress response pathway sensor CHEK1 and induction of apoptosis)	Human colon cancer cell lines HCT-116 (p53+/+ and p53−/−)	ND	[57]
	Active (ND)	Ehrlich ascites carcinoma bearing mice Ehrlich ascites carcinoma bearing mice	50 mg/L 5 mg/kg	[53] [54]
	Active (downregulation of NF-*κ*B)	Orthotopic model of pancreatic cancer (*in vitro*) Orthotopic model of pancreatic cancer (*in vivo*)	ND 3 mg/mouse	[55] [86]
	Active (antioxidant activity)	Fibrosarcoma induced by 20-methylcholanthrene (MC) in male Swiss albino mice (*in vitro*) Swiss albino mice (*in vivo*)	ND 0.01% in drinking water	[59] [60]
	Active (antioxidant activity)	Osteoblasts cells (MG 63) in tissue culture	32–64%^∗,b^	[61]

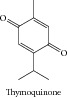	Active (induction of p53-independent apoptosis)	Human osteosarcoma cells (p53-null MG63 cells) (p53-mutant MNNG/HOS cells)	17 *µ*M 38 *µ*M	[62]
	Active (inhibition of NF-*κ*B and antiangiogenesis effect)	Osteosarcoma (*in vitro*) Osteosarcoma (*in vivo*)	ND 6 mg/kg	[63]
	Active (induction of apoptosis via p53-dependent pathway)	HeLa (epithelioid carcinomic cell line)	2.80–5.93 mg/mL∗	[64]

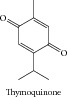	Active (involvement of reactive oxygen species and activation of ERK and JNK signaling)	Caco-2 (human colon cancer cell)	12.5–15.0 *µ*M∗	[65]
		HCT-116 (human colon cancer cell)	14–30 *µ*M∗	
		LoVo (human colon cancer cell)	28–38 *µ*M∗	
		DLD-1 (human colon cancer cell)	23–42 *µ*M∗	
		HT-29 (human colon cancer cell)	110 *µ*M	
	Active (binding to bovine serum albumin)	DLD-1 (human colon cancer cell)	ND	[88]
		HCT-116 (human colon cancer cell)	ND	
	Active (ND)	A549 (human nonsmall cell lung cancer (NSCLC) cell line)	ND	[67]
	Active (antioxidant activity)	ES-2 (ovarian cancer cell line)	ND	[66]
	Active (prooxidant cytotoxic mechanism)	Prostate cancer cell lines	ND	[68]
	Active (ND)	66 cl-4-GFP (resistant mouse mammary gland cell line) *in vivo*	10 mg/kg	[69]
	Active (disruption in cell-cycle checkpoints)	LNCaP (prostate cancer cell line) LNCaP (prostate cancer cell line )	ND ND	[70] [71]
	Active (induction of apoptosis)	p53-null myeloblastic leukemia HL 60 cells	23 *µ*M	[72]

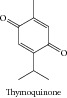	Active (increase of ROS generation and decreased GSH levels)	Androgen receptor (AR) independent (C4-2B)	100 *μ*mol/L	[73]
		AR naive (PC-3) prostate cancer cells	86 *μ*mol/L	
	Active (cell cycle arrest; induction of apoptosis)	COS31 (canine osteosarcoma)	ND	[74]
	Active (cell cycle arrest; induction of apoptosis)	HepG2 (hepatocellular carcinomic human cell line)	350 *µ*M	[75]
	Active (cell cycle arrest, increase in the expression of the protein p53 and decrease in cyclin B1 protein)	Primary mouse keratinocytes, SP-1 (papilloma) I7 spindle carcinoma cells	30 *µ*M 60 *µ*M	[76]
	Active (inhibition of telomerase)	Human glioblastoma cells	ND	[77]
	Active (inhibition of PDE1A expression)	Jurkat cell (acute lymphoblastic leukemia cell line)	ND	[79]
	Active (downregulated MUC4 expression and induction of apoptosis)	The MUC4-expressing pancreatic cancer cells FG/COLO357 CD18/HPAF	73 *μ*mol/L 73 *μ*mol/L	[80]

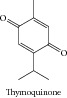	Active (upregulation of PTEN expression and induction of apoptosis)	Doxorubicin-resistant human breast cancer MCF-7/DOX cell	35–70%^a^	[82]
	Active (ND)	Parental and multidrug resistant (MDR) human tumor cell lines	78 *µ*M	[81]
	Active (thymoquinone-loaded nanoparticles activity)	MDA-MB-231 (human metastatic breast cancer cell line)	ND	[84]
	Active (comparison of thymoquinone versus thymoquinone-loaded nanoparticles activities)	HCT-116 (colon cancer cell line)	15% versus 85%^b^	[83]
		MCF-7 (breast cancer cell line)	30% versus 88%^b^	
		PC-3 (prostate cancer cell line)	30% versus 85%^b^	
		U-266 (multiple myeloma cell line)	55% versus 70%^b^	
		HCT116 (colon cancer cell line)	24%^c^	
	Activity of derivatives of thymoquinone	HCT116p53−/− colon cancer	72%^c^	[85]
		HepG2 (hepatocellular carcinomic human cell line)	75%^c^	
		HL-60 (acute promyelocytic leukemia cells)	0.13–>100 *µ*M∗	
	Activity of analogs of thymoquinone	518A2 (melanoma cell line)	3.9–>100 *µ*M∗	[87]
		multidrug-resistant KB-V1/Vbl cervix	7.0–79.9 *µ*M∗	
		MCF-7/Topo (breast carcinoma)	2.8–>100 *µ*M∗	

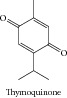	Active (ND)	Mouse Ehrlich ascites carcinoma tumor	ND	[89]
	Active (inhibition of Akt phosphorylation; induction of apoptosis; inhibition of HDAC2 proteins)	LNM35 (human lung cancer cell)	50–78 *µ*M∗	
		HepG2 (human hepatoma cell)	34 *µ*M	
		HT29 (human colorectal cancer cell)	50–78 *µ*M∗	[96]
		MDA-MB-435 (human mammary adenocarcinoma cell)	50–78 *µ*M∗	
		MDA-MB-231 (human mammary adenocarcinoma cell)	50–78 *µ*M∗	
		MCF-7 (human mammary adenocarcinoma cell)	50–78 *µ*M∗	
	Active (ND)	*In vivo* activity of quinone reductase and glutathione transferase in mice liver	1, 2 or 4 mg/kg	[90]
	Active (cell cycle arrest; induction of apoptosis via Akt modulation)	MDA-MB-468 (human mammary adenocarcinoma)	12.30 *µ*M	[92]
		T-47D (human mammary ductal carcinoma)	18.06 *µ*M	
	Active (induction of apoptosis)	HL-60 (human promyelocytic leukemia cell)	27.8 *µ*M	[93]
		518A2 (melanoma cell line)	28.3 *µ*M	

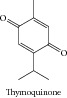		HT-29 (colon carcinoma cell)	46.8 *µ*M	
	Active (induction of apoptosis)	KB-V1 (cervix carcinoma cell)	25.8 *µ*M	[93]
		MCF-7 (human mammary adenocarcinoma cell)	20.1 *µ*M	
		Multidrug-resistant variants	18.7–57.2 *µ*M∗	
		MCF-7 (breast cancer cell line)	32–48 *µ*M∗	
	Active (modulation of the PPAR-*γ* activation pathway)	MDA-MB-231 (breast cancer cell line)	11–24 *µ*M∗	[193]
		BT-474 (breast cancer cell line)	18–38 *µ*M∗	
	Active (condition T cells *in vitro* for adoptive T cell therapy against cancer and infectious disease)	OT-1 (transgenic CD8+) T cells	ND	[95]
	Active (induction of apoptosis)	Mouse model of familial adenomatous polyposis	375 mg/kg	[97]
	Active (comparison of thymoquinone and thymoquinone in liposomes effects)	MCF-7 (breast cancer cell line) T47D (breast cancer cell line)	40 *µ*M versus 200 *µ*M 15 *µ*M versus 75 *µ*M	[99]
		A431 (human epidermoid carcinoma)	10 *µ*M	
	Active (cell cycle arrest and induction of apoptosis)	Hep-2 (larynx epidermoid carcinoma)	10 *µ*M	[100]
		Sarcoma 180 *in vivo *	10 mg/kg	

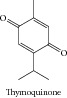	Active (antimicrotubule drug)	U87 (human astrocytoma cell line-solid tumor model)	ND	[101]
		Jurkat cells (acute lymphoblastic leukemia cell line)	ND	

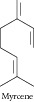	Active	Mouse P388 leukemia cell	ND	[17]
		HeLa (human cervical carcinoma)	>200 *µ*g/mL	
	Active (ND)	A-549 (human lung carcinoma)	>200 *µ*g/mL	[103]
		HT-29 (human colon adenocarcinoma) cell lines	>200 *µ*g/mL	
		Crown gall tumors	50%^b^	
	Active (ND)	MCF-7 (breast carcinoma)	<10^–2^ mug/mL	[102]
		HT-29 (colon adenocarcinoma)	<10^–2^ mug/mL	
		A-549 (lung carcinoma)	<10^–2^ mug/mL	
	Active (cell cycle arrest and induction of apoptosis)	HepG2 (hepatocellular carcinomic human cell line)	9.23 *µ*g/mL	[9]
		B16F10 (murine melanoma)	12.27 *µ*g/mL	

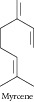	Active (cell cycle arrest and induction of apoptosis)	B16F10 (murine melanoma) K562 (erythromyeloblastoid leukemia cell line)	12.27 *µ*g/mL ND	[9]

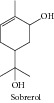	Active (induction of the hepatic detoxification enzymes glutathione S-transferase GST and uridine diphosphate-glucuronosyltransferase UDPGT)	DMBA-induced rat mammary carcinogenesis	ND	[104]

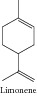	Active (antitumorigenic effects induction of apoptosis)	Pancreatic, mammary, and prostatic tumors	ND	[105]
	Active (inhibition of the isoprenylation of small G proteins)	DMBA- and ^5^NMU-induced rat mammary carcinomas	10% in diet	[114]
	Active (combined limonene and 4-hydroxyandrostrenedione)	NMU-induced rat mammary carcinomas	5% limonene and 4-hydroxyandrostrenedione (12.5 mg/kg)	[115]
	Active (ND)	DMBA-induced rat mammary carcinogenesis	ND	[116]
	Active (induction of apoptosis and antiangiogenic effect)	SW480 (human colorectal adenocarcinoma)	ND	[117]

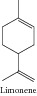	Active (induction of apoptosis and antiangiogenic effect)	HT-29 (colon adenocarcinoma)	ND	[117]
	Active (immunomodulatory effect)	BW5147 (murine T cell lymphoma)	35 *µ*g/mL	[118]
	Active (ND)	B16F-10 (melanoma cells in mice)	100 *µ*M/kg	[119]
	Active (induction of the hepatic detoxification enzymes glutathione S-transferase GST and uridine diphosphate-glucuronosyltransferase UDPGT)	DMBA-induced rat mammary carcinogenesis	ND	[104]
	Activity of derivatives limonene (inhibition of protein prenylation)	*In vitro* assays with mammalian and yeast farnesyltransferase (PFT) and geranylgeranyltransferase (PGGT) proteins	ND	[107]
	Active (inhibition of NNK metabolic activation)	NNK-induced lung tumorigenesis in mice	183 *µ*mol	[120]
	Active (ND)	AflatoxinB1-induced hepatocarcinogenesis	5% in diet	[121]
	Active (induction of apoptosis)	K562 (erythromyeloblastoid leukemia cell line) HL-60 (acute promyelocytic leukemia cells)	ND ND	[110]

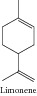	Active (ND)	Colonic carcinogenesis in rats	5%	[111]
	Active (high affinity with HMG-CoA reductase)	*in silico* approaches	ND	[112]
	Active (increase of GST activity)	Several tissues of female A/J mice	20 mg/0.3 mL of the oil	[122]
		MCF-7 (human breast adenocarcinoma)	14 *µ*g/mL	
	Active (ND)	K562 (erythromyeloblastoid leukemia cell line)	16 *µ*g/mL	[106]
		PC12 (rat adrenal pheochromocytoma cell line)	>100 *µ*g/mL	
		A-549 (lung carcinoma)	<10^–2^ mug/mL	
	Active (ND)	MCF-7 (breast carcinoma)	<10^–2^ mug/mL	[102]
		HT-29 (colon adenocarcinoma)	<10^–2^ mug/mL	
		A-549 (lung carcinoma)	ND	
	Active (effect on gap junction intercellular communication)	W1-38 (human fibroblast lung normal cells)	ND	[113]
		CACO2 (human colorectal adenocarcinoma)	ND	

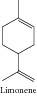	Active (effect on gap junction intercellular communication)	PaCa (human pancreatic carcinoma cells)	ND	[113]
	Active (ND)	A-549 (lung carcinoma)	0.098 *μ*L/mL	[108]
		HepG2 (hepatocellular carcinomic human cell line)	0.150 *μ*L/mL	
	Active (ND)	Molecular docking	ND	[109]
	Active (involvement of c-myc oncoprotein)	NDEA induced hepatocarcinogenesis	5% in diet	[123]

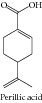		HTB-43 (human head and neck squamous cell carcinoma cell line)	10%^c^	
	Active (cell cycle arrest; induction of apoptosis)	SCC-25 (human head and neck squamous cell carcinoma cell line)	19%^c^	[126]
		BroTo (human head and neck squamous cell carcinoma cell line)	9%^c^	
	Active (cell cycle arrest; induction of apoptosis)	A549 (human lung adenocarcinoma) H520 (squamous lung cell carcinoma)	3.6 mM ND	[125]

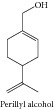	Active (induction of apoptosis)	9,10-Dimethylbenz(a)anthracene (DMBA)-initiated and 12-O-tetradecanoylphorbol-13-acetate (TPA)-promoted skin tumorigenesis	6 or 12 mg/kg	[146]
	Active (cell cycle arrest; induction of apoptosis)	Advanced rat mammary carcinomas	0.1 g/kg	[147]
	Active (cell cycle arrest; induction of apoptosis)	Bcr/Abl-transformed leukemia cells	ND	[148]
	Active (cell cycle arrest and induction of apoptosis)	Bcr/Abl-transformed myeloid cell lines	300–400 *µ*m	[139]
	Active (ND)	Hamster pancreatic tumors	1.2–2.4 g/kg	[127]
	Active (ND)	MIA PaCa2 (human pancreatic tumor cells)	60–90%^2^	[128]
		PC-1 (hamster pancreatic adenocarcinoma)	40 g/kg	
	Active (induction of Bak-induced apoptosis)	B12/13 (pancreatic adenocarcinoma cell line)	150 *µ*M	[130]
	Active (inhibition of the prenylation of growth-regulatory proteins)	Pancreatic adenocarcinoma cells	ND	[129]

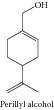		AsPC-1 (pancreatic adenocarcinoma cell line)	300 *µ*mol/L	
	Active (induction of apoptosis)	MIA PaCa-2 (pancreatic adenocarcinoma cell line)	350 *µ*mol/L	[131, 149]
		PANC-1 (pancreatic adenocarcinoma cell line)	350 *µ*mol/L	
		BxPC-3 (pancreatic adenocarcinoma cell line)	550 *µ*mol/L	
	Active (induction of apoptosis)	K562 (erythromyeloblastoid leukemia cell line)	81.0 *µ*mol/L	[133]
	Active (antiangiogenic activity)	BLMVECs (bovine lung microvascular endothelial cells)	ND	[135, 136]
	Active (c-Myc-dependent apoptosis)	Bcr/Abl-transformed leukemia cells	ND	[134]
	Active (cell cycle arrest; induction of apoptosis)	A549 (human lung adenocarcinoma epithelial cell line) H520 (squamous cell carcinoma)	1.4 mM 1.7 mM	[125]
	Active (inhibition of Na^+^/K^+^-ATPase activity)	Guinea pig brain and kidney were used in the preparation of homogenates and Na/K-ATPase-enriched fractions	1.0 mM for the brain enzyme and 1.5 mM for the kidney enzyme	[137]

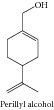	Active (inhibition of protein isoprenylation and cell proliferation)	HT-29 (colon adenocarcinoma cell)	ND	[138]
	Active (modulation of the expression of AP-1 target genes)	Breast cancer cells	ND	[140]
	Active (antitumor effect potentiated by hyperthermia)	SCK mammary carcinoma cells of A/J mice	20–58%^a^	[141]
	Active (inhibition of ubiquinone synthesis and block of the conversion of lathosterol to cholesterol)	NIH3T3 (mouse fibroblast cell line)	ND	[142]
	Active (activity of metabolites of perillyl alcohol)	Inhibition of protein prenyltransferases *in vitro *	1 mM	[107]
	Active (inhibition of the *in vivo* prenylation of specific proteins)	NIH3T3 (mouse fibroblast cell line)	0.5 or 1.0 mM	[143]
	Active (ND)	AflatoxinB1-induced hepatocarcinogenesis	2% in diet	[121]
	Active (antimetastatic activity)	C6 (glial cell line)	ND	[144]
	Active (phases I/II study)	Human malignant gliomas	0.3% v/v	[145]
	Active (pretreated before exposure to radiation)	HTB-43 (head and neck squamous cell carcinoma cell line)	71%^b^	[126]

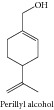	Active (pretreated before exposure to radiation)	SCC-25 (head and neck squamous cell carcinoma cell line)	68%^b^	[126]
		BroTo (head and neck squamous cell carcinoma cell line)	53%^b^	
	Active (radio-/Chemosensitizer)	Glioma cells	ND	[152]
	Active (cell cycle arrest; induction of apoptosis)	PC12 (rat adrenal pheochromocytoma cell line)	ND	[150]
	Active (pharmacokinetics studies)	Pharmacokinetics studies in dogs	ND	[151]
	Active (phase I)	Human advanced malignancies	800–2400 mg/m^2^/dose	[153]
	Active (telomerase activity)	Prostate cancer cells	ND	[132]

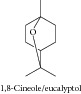	Active (ND)	HepG2 (hepatocellular carcinomic human cell line); HeLa (human cervical carcinoma); MOLT-4 (human lymphoblastic leukemia T cell line); K-562 (human chronic myelogenous leukemia cell line); CTVR-1 (an early B cell line from the bone marrow cells of a patient with acute myeloid leukemia)	ND	[14]

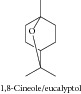	Active (ND)	Melanoma H157 cells Carcinoma HT14 cells	3.4–95.3%^2^ 5.7–96.2%^2^	[159]
	Active (induction of apoptosis)	KB (human papilloma cell line)	ND	[160]
		SK-OV-3 (human ovarian adenocarcinoma)	1.10‰ (v/v)	
	Active (ND)	HO-8910 (human epithelial ovarian cancer)	2.90‰ (v/v)	[158]
		Bel-7402 (human hepatocellular carcinoma)	3.47‰ (v/v)	
	Active (binds to the Caspase 3)	Molecular docking studies	ND	[109]

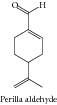		MCF-7 (breast carcinoma)	ND	
	Active (ND)	K-562 (human chronic myelogenous leukemia cell line)	ND	[106]
		PC-12 (rat adrenal gland pheochromocytoma)	ND	

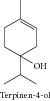		HepG2 (hepatocellular carcinomic human cell line)	ND	
		HeLa (epithelioid carcinomic cell line)	ND	
	Active (ND)	MOLT-4 (human lymphoblastic leukemia T-cell line)	ND	[14]
		K-562 (human chronic myelogenous leukemia cell line)	ND	
		CTVR-1 (early B cell line from the bone marrow cells of a patient with acute myeloid leukemia)	ND	
	Active (induction of apoptosis)	Human melanoma M14 WT cells and their resistant variants	ND	[161]
	Active (ND)	Drug-sensitive and drug-resistant melanoma cells	ND	[162]
	Active (cell cycle arrest and induction of necrosis)	AE17 (mesothelioma murine cancer cells) B16 (melanoma cells)	0.01–0.02 0.04–0.05	[163]
	Active (ND)	A-549 (lung carcinoma); DLD-1 (human colorectal adenocarcinoma)	>100 *µ*M >100 *µ*M	[50]

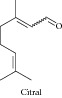	Active (ND)	P388 mouse leukemia cells	ND	[164]
	Active (ND)	MCF-7 (breast carcinoma)	92.3%^c^	[165]
	Active (induction of apoptosis-activating p53)	Endometrial cancer cell lines Ishikawa and ECC-1 (endometrial carcinoma cell line)	2.3 *µ*g/mL	[166]
	Active (induction of apoptosis)	NB4 (acute promyelocytic leukemia cell line)	3.995 *µ*g/mL	[167]

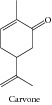	Active (induction of apoptosis)	Hep-2 (larynx epidermoid carcinoma)	0.47–0.62 mM∗	[35]
	Active (ND)	HeLa (human cervical carcinoma)	74.5 *µ*g/mL	[168]
	Active (induction of oxidative stress)	Cultured primary rat neuron and N2a neuroblastoma (NB) cells	ND	[169]
	Active (ND)	MCF-7 (breast carcinoma)	0.63 *μ*M	[46]
		K-562 (human chronic myelogenous leukemia cell line)	0.17 *μ*M	
		P-815 (mouse lymphoblast like mastocytoma cell line)	0.16 *μ*M	
		CEM (human acute lymphoblastic leukemia)	0.11 *μ*M	

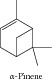	Active (oxidative stress and reporter gene activities of antioxidant response element (ARE), activator protein 1 (AP-1), and nuclear factor NF-*κ*B)	A549 (lung carcinoma) HepG2 (hepatocellular carcinomic human cell line)	250 ppm 203 ppm	[174]
	Active (immunomodulatory effect)	BW 5147 (murine T cell lymphoma) Normal murine lymphocytes	1100 *μ*g/mL 72 *μ*g/mL	[118]
		HepG2 (hepatocellular carcinomic human cell)	1393.3 *µ*g/mL	
		K562 (human chronic myelogenous leukemia cell line)	679.1 *µ*g/mL	
	Active (ND)	H-460 (lung large cell carcinoma)	501.8 *µ*g/mL	[170]
		N-87 (gastric carcinoma)	840.6 *µ*g/mL	
		SW-620 (colon adenocarcinoma)	786.2 *µ*g/mL	
	Active (ND)	Dual reverse virtual screening protocol	ND	[109]
	Active (ND)	SK-OV-3 (human ovarian adenocarcinoma) HO-8910 (human epithelial ovarian cancer)	0.052‰ (v/v) 0.11‰ (v/v)	[158]

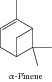	Active (ND)	Bel-7402 (human hepatocellular carcinoma)	0.32‰ (v/v)	[158]
	Active (ND)	MCF-7 (mammary adenocarcinoma) MDA-MB-231 (mammary adenocarcinoma) MDA-MB-468 (mammary adenocarcinoma) UACC-257 (malignant melanoma)	64.3 *µ*g/mL^5^ >100 *µ*g/mL^5^ 27.7 *µ*g/mL^5^ 13.5 *µ*g/mL^5^	[171]
	Active (induction of apoptosis)	U937 (histiocytic lymphoma cells)	ND	[172]
		HeLa (human cervical carcinoma)	172.7 *μ*g/mL	
	Active (ND)	A-549 (human lung carcinoma)	183.2 *μ*g/mL	[103]
		HT-29 (human colon adenocarcinoma)	>200 *μ*g/mL	
		MCF-7 (human breast carcinoma cell line)	20.6 *µ*g/mL	
	Active (ND)	MDA-MB-468 (human breast carcinoma cell line)	39.2 *µ*g/mL	[173]
		UACC-257 (human breast carcinoma cell line)	16.3 *µ*g/mL	

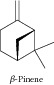	Active (ND)	A-549 (lung carcinoma) DLD-1 (human colorectal adenocarcinoma)	85.0 *μ*M >100 *μ*M	[50]
		MCF-7 (breast carcinoma)		
	Active (ND)	A375 (human melanoma)	ND	[170]
		HepG2 (hepatocellular carcinomic human cell line)		
	Active (immunomodulatory effect)	BW5147 (murine T cell lymphoma)	114.81 *μ*g/mL	[118]
		MCF-7 (breast carcinoma)	176.5–242.6 mM∗	
	Active (ND)	A375 (human melanoma)	198.5–264.7 *μ*M∗	[91]
		HepG2 (hepatocellular carcinomic human cell)	147.1–198.5 *μ*M∗	
	Active (ND)	MCF-7 (human breast carcinoma cells) MDA-MB-231 (human breast carcinoma cells) MDA-MB-468 (human breast carcinoma cells) UACC-257 (human breast carcinoma cells)	ND ND ND ND	[171]
	Active (ND)	Dual reverse virtual screening protocol	ND	[109]

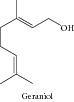	Active (cell cycle arrest)	(Caco-2) human colon cancer cell line	70%^a^	[179]
	Active (blockade of the morphological and functional differentiation of the cells)	Human colonic cancer cells	30%^c^	[175]
		Caco-2 (human epithelial colorectal adenocarcinoma cells)	250 *µ*M	
	Active (thymidylate synthase and thymidine kinase expression)	SW620 (human colon adenocarcinoma)	330 *µ*M	[176]
		TC-118 (colorectal tumor)	150 mg/kg	
	Active (ND)	Human MIA PaCa2 pancreatic tumor cells and hamster (transplanted PC-1 pancreatic adenocarcinomas)	60–90%^a^ 40 g/kg diet	[128]
	Active (effects on mevalonate and lipid metabolism)	HepG2 (hepatocellular carcinomic human cell line)	≥90%^a^	[177]
	Active (high affinity with HMG-CoA reductase)	HepG2 (hepatocellular carcinomic human cell line)	ND	[178]
	Active (high affinity with HMG-CoA reductase)	*In silico* approaches	ND	[112]
	Active (activity of the detoxifying enzyme glutathione S-transferase)	Mucosa of the small intestine and large intestine	ND	[122]

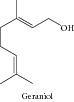	Active (induction of apoptosis)	Hepatocarcinogenesis in rats	25 mg/100 g	[180]
	Active (induction of apoptosis; inhibition of RhoA activation)	Hepatocarcinogenesis in rats	25 mg/100 g	[181]
	Active (ND)	MIA PaCa2 (human pancreatic tumor cells) PC-1 (hamster pancreatic adenocarcinoma)	60–90%^a^ 40 g/kg diet	[128]
	Active (nuclear factor erythroid 2-related factor-2 (Nrf2) activation)	4NQO-induced oral carcinogenesis in mouse	200 mg/kg	[182]
	Active (ND)	Dual reverse virtual screening protocol	ND	[109]

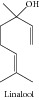	Active (inhibition of mitochondrial complexes I and II, increase of ROS and decrease of ATP and GSH levels)	HepG2 (hepatocellular carcinomic human cell line)	0.4–2 *µ*M	[187]
	Active (ND)	C32 (amelanotic melanoma cell line)	23.2 *µ*g/mL	[94]
	Active (ND)	HeLa (human cervical carcinoma cells) AGS (stomach carcinoma cells) BCC-1/KMC (skin carcinoma cells) H520 (lung carcinoma cells)	0.37 *µ*g/mL 14.1 *µ*g/mL 14.9 *µ*g/mL 21.5 *µ*g/mL	[184]

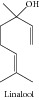	Active (ND)	U_2_OS (bone carcinoma cells)	21.7 *µ*g/mL	[184]
	Active (ND)	U937 (histiocytic lymphoma cell line) P3HR1 (Burkitt lymphoma cell line)	3.51 *µ*g/mL 4.21 *µ*g/mL	[185]
	Active (induction of apoptosis by activation of p53 and CDKIs)	Kasumi-1 (acute myeloid leukemia)	49.53–127.14 *µ*M	
		HL-60 (acute myeloid leukemia)	49.53–127.14 *µ*M	
		THP-1 (acute myeloid leukemia)	>144.04 *µ*M	
		U937 (acute myeloid leukemia)	>144.04 *µ*M	
		KG-1 (acute myeloid leukemia)	>144.04 *µ*M	
		NB4 (acute myeloid leukemia)	>144.04 *µ*M	[188]
		K562 (blast crisis of chronic myeloid leukemia)	>144.04 *µ*M	
		Molt-4 (acute T-lymphoblastic leukemia)	49.53–127.14 *µ*M	
		H-9 (acute T-lymphoblastic leukemia)	ND	
		Jurkat (acute T-lymphoblastic leukemia)	>144.04 *µ*M	
		Raji (human Burkitt's lymphoma)	49.53–127.14 *µ*M	
		L428 (Hodgkin's lymphoma)	>144.04 *µ*M	

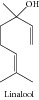	Active (potentiate doxorubicin-induced cytotoxicity; induction of apoptosis)	MCF7 WT (human breast adenocarcinoma)	0.62–0.79 *µ*M	[189]
		multidrug resistant MCF7 Adr^R^	1.24–3.0 *µ*M	
	Active (ND)	C32 (amelanotic melanoma cell line)	23.2 *μ*g/mL	[186]
		Renal adenocarcinoma cells	23.8 *μ*g/mL	
	Active (induction of apoptosis; promotion cell differentiation)	HL-60 (acute promyelocytic leukemia cells)	ND	[190]
	Active (combination of doxorubicin-linalool increased doxorubicin influx in tumor cells)	Mouse P388leukemia cells (*in vitro*)	ND	[191]
		Mouse P388leukemia cells (*in vivo*)	1.0 mg/kg/day	

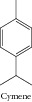	Active (ND)	A-549 (lung carcinoma) DLD-1 (colorectal adenocarcinoma)	43.0 *µ*g/mL 46.0 *µ*g/mL	[50]

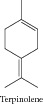	Active (ND)	MCF-7 (human breast cancer cells)	1.3 mg/L	[192]

ND: not determined.

∗Variable values refer to differences on the concentrations used, time of treatment, cell line, and/or assay used.

^a^% survival and/or proliferation.

^b^% growth inhibition.

^c^% mortality.

## Abbreviations

DMBA: 7,12-Dimethylbenz[*α*]anthracene
DEN: Diethylnitrosamine
ROS: Reactive oxygen species
GSH: Reduced glutathione
NMU: N-Nitroso-N-methylurea
NNK: 4-(Methylnitrosamino)-1-(3-pyridyl)-1-butanone
HMG-CoA: 3-Hydroxy-3-methylglutaryl-CoA
GST: Glutathione S-transferase
NDEA: N-Nitrodiethylamine.
